# Ramucirumab plus paclitaxel as switch maintenance in patients with advanced HER2-negative gastric or gastro-oesophageal junction cancer: a cost-effectiveness analysis

**DOI:** 10.3389/fphar.2025.1616826

**Published:** 2025-09-12

**Authors:** Jiefeng Luo, Zhengxiong Li, Qiong Du, Jiyong Liu

**Affiliations:** ^1^ Department of Pharmacy, Fudan University Shanghai Cancer Center, Shanghai, China; ^2^ Department of Oncology, Shanghai Medical College, Fudan University, Shanghai, China; ^3^ School of Medical Informatics and Engineering, Xuzhou Medical University, Xuzhou, China

**Keywords:** ramucirumab, paclitaxel, gastric cancer, gastro-oesophageal junction cancer, cost-effectiveness

## Abstract

**Objectives:**

The ARMANI trial demonstrated that ramucirumab plus paclitaxel (switch maintenance group) significantly prolonged progression-free survival (PFS) and overall survival in patients with advanced HER2-negative gastric cancer (GC) and gastroesophageal junction cancer (GEJC) compared to continued first-line oxaliplatin-based chemotherapy (control group). However, its cost-effectiveness remained unclear. This study aimed to evaluate its cost-effectiveness from the Chinese and United States (US) healthcare system perspective.

**Methods:**

A partitioned survival model was developed to compare the total costs, quality-adjusted life years (QALYs) and incremental cost-effectiveness ratios (ICERs) of switch maintenance group versus control group over a 10-year time horizon. Survival data were sourced from the ARMANI trial. Cost and utility were derived from open-access databases and published literature. The robustness of the results was verified through one-way sensitivity analysis and probabilistic sensitivity analysis (PSA). Additionally, subgroup analysis and scenario analysis were conducted.

**Results:**

The switch maintenance group yielded incremental gains of 0.15 QALYs in China and 0.16 QALYs in the US, with corresponding incremental costs of $56,738.32 and $185,250.55, resulting in ICERs of $373,219.84/QALY and $1,193,220.74/QALY, respectively. For the PD-L1 CPS ≥5 subgroup, incremental QALYs increased to 0.24 and 0.25, with incremental costs rising to $62,741.24 and $206,107.13, yielding ICERs of $266,259.94/QALY and $835,740.90/QALY, respectively. One-way sensitivity analysis revealed that the utility of PFS, the price of ramucirumab, and patient body weight were the most influential factors on the ICER, with consistent results observed from both Chinese and US perspectives. To be cost-effective in a 50% of chance, ramucirumab would need to reduce its price to 14.2% of the original price ($0.743 per mg) in China and 13.92% ($2.088 per mg) in the US, respectively.

**Conclusion:**

Ramucirumab plus paclitaxel is unlikely to be cost-effective compared to continuing oxaliplatin-based chemotherapy for patients with advanced HER2-negative GC or GEJC in China and US.

## Introduction

Gastric cancer (GC), including gastroesophageal junction cancer (GEJC), is a major global health burden (Cao et al., 2020). It ranks as the fifth most diagnosed malignancy and the third leading cause of cancer-related mortality worldwide. Approximately 1 million new cases are reported each year, with the majority occurring in east Asia (Smyth et al., 2020). China accounts for 44% of the global GC incidence (He et al., 2024). In the United States (US), the incidence and mortality rates of GC have been steadily declining (Thrift and El-Serag, 2020). However, GC exhibits the lowest 5-year overall survival (OS) rate among all cancers, posing significant implications for the US healthcare system (Thrift and El-Serag, 2020). Due to the high proportion of late-stage diagnoses, the prognosis for advanced GC remains poor (Smyth et al., 2020), with a five-year survival rate of less than 10% in metastatic cases (Yang et al., 2020).

Beyond its clinical impact, GC imposes a heavy financial burden. In China, newly diagnosed patients face an average annual out-of-pocket expense of $5,368, consuming 63.80% of their household income from the preceding year (Zhang et al., 2020). Despite improved therapeutic efficacy in GC treatment, the associated healthcare burden and costs have risen substantially in US, with average hospital expenses surging from $75,341 per patient in 2003 to $91,385 per patient in 2014 (Liu et al., 2018).

The standard first-line treatment for advanced GC includes a fluoropyrimidine combined with a platinum-based chemotherapy (National Comprehensive Cancer Network, 2024; Lordick et al., 2022). Vascular Endothelial Growth Factor (VEGF) and Vascular Endothelial Growth Factor Receptor-2 (VEGFR-2) mediated signaling and angiogenesis contribute to the pathogenesis of gastric cancer (Wang et al., 2024). Inhibition of VEGFR-2 has been demonstrated to reduce tumor growth and neovascularization (Jung et al., 2002). Ramucirumab, a human IgG1 monoclonal antibody VEGFR-2 antagonist, blocks ligand binding and receptor-mediated pathway activation in endothelial cells (Coudert et al., 2012; Hu et al., 2019). Based on the great progress made in the RAINBOW trial, ramucirumab plus paclitaxel was approved by Food and Drug Administration (FDA) for the second-line treatment of advanced GC (Wilke et al., 2014). The Chinese Society of Clinical Oncology (CSCO) guidelines (Wang et al., 2024) and National Comprehensive Cancer Network (NCCN) guidelines (National Comprehensive Cancer Network, 2024) recommend ramucirumab plus paclitaxel as the preferred second-line treatment for advanced Human Epidermal Growth Factor Receptor 2 (HER2)-negative GC. However, despite the significant advances demonstrated in the RAINBOW trial, the median OS remains less than 10 months (Wilke et al., 2014). Switch maintenance therapy, which involves an early transition to a non-cross-resistant regimen (Lee and Chung, 2014), has shown clinical benefits in other cancers such as non-small cell lung cancer (Coudert et al., 2012; Hu et al., 2019). Therefore, ramucirumab plus paclitaxel as switch maintenance or early second-line therapy for patients with HER2-negative GC who have received standard fluoropyrimidine combined with a platinum-based chemotherapy induction deserves further study.

The phase III ARMANI trial (NCT02934464) evaluated ramucirumab plus paclitaxel as a switch maintenance therapy (switch maintenance group) compared to continuing first-line oxaliplatin-based chemotherapy (control group). The study showed significant improvements in clinical outcomes. The switch maintenance group had longer median progression-free survival (PFS) (8.8 vs. 6.1 months; Hazard ratio [HR] = 0.61) and OS (15.8 vs. 12.7 months; HR = 0.75). Additionally, the disease control rate was higher (85% vs. 54%) (Randon et al., 2024).

Although ramucirumab plus paclitaxel has shown promising clinical benefits, the high cost of ramucirumab raises concerns about affordability, especially in resource-limited settings (Saito et al., 2017; Li et al., 2020). Pharmacoeconomic studies can help clinical decision makers and patients allocate resources rationally and improve the overall efficiency of medical resource allocation (Liu et al., 2020). However, no cost-effectiveness analysis has assessed ramucirumab plus paclitaxel as a first-line switch maintenance therapy. Therefore, this study aimed to evaluate the cost-effectiveness of ramucirumab plus paclitaxel as first-line switch maintenance therapy or early second-line treatment for GC from the perspective of the Chinese and US healthcare system.

## Materials and methods

This study was conducted in accordance with the Consolidated Health Economic Evaluation Reporting Standards (CHEERS 2022) Statement (Supplementary Table S1) (Husereau et al., 2022). Institutional review board approval or patient consent was not required as this analysis utilized publicly available data and modeling techniques.

### Patients and interventions

Our study simulated a patient population aligned with the ARMANI trial. These patients had histologically confirmed advanced HER2-negative gastric or gastro-oesophageal junction cancer, with an Eastern Cooperative Oncology Group (ECOG) performance status of 0 or 1. All patients were assumed to have achieved disease control after 3 months of first-line FOLFOX (folinic acid, fluorouracil, oxaliplatin) or CAPOX (capecitabine, oxaliplatin) therapy. For the Chinese perspective, patient weight was 69 kg with a body surface area (BSA) of 1.74 m^2^; for the US perspective, weight was 75 kg with a BSA of 1.80 m^2^. Other key baseline characteristics mirrored those of patients in the ARMANI study.

Patients were randomly assigned to the switch maintenance group or the control group. Patients assigned to the switch maintenance group received intravenous ramucirumab 8 mg/kg on days 1 and 8, and paclitaxel 80 mg/m^2^ on days 1, 8, and 15, once every 28 days until disease progression, unacceptable toxicity, withdrawal of informed consent, or patient death. Patients in the control group continued to receive FOLFOX or CAPOX and received fluorouracil therapy alone (capecitabine or 5-fluorouracil) after a maximum of 24 weeks of treatment. After disease progression, 58% of patients in the switch maintenance group and 56% of patients in the control group received subsequent systemic therapy (Randon et al., 2024). Based on the CSCO guidelines (Wang et al., 2024) and the NCCN guidelines (National Comprehensive Cancer Network, 2024), intravenous injection of irinotecan 150 mg/m^2^ on the first day of a 21-day cycle as the subsequent treatment for patients was chosen for disease progression. For patients who did not receive antitumor systemic therapy after disease progression, we assumed they received best supportive care (BSC).

### Model structure

**FIGURE 1:**
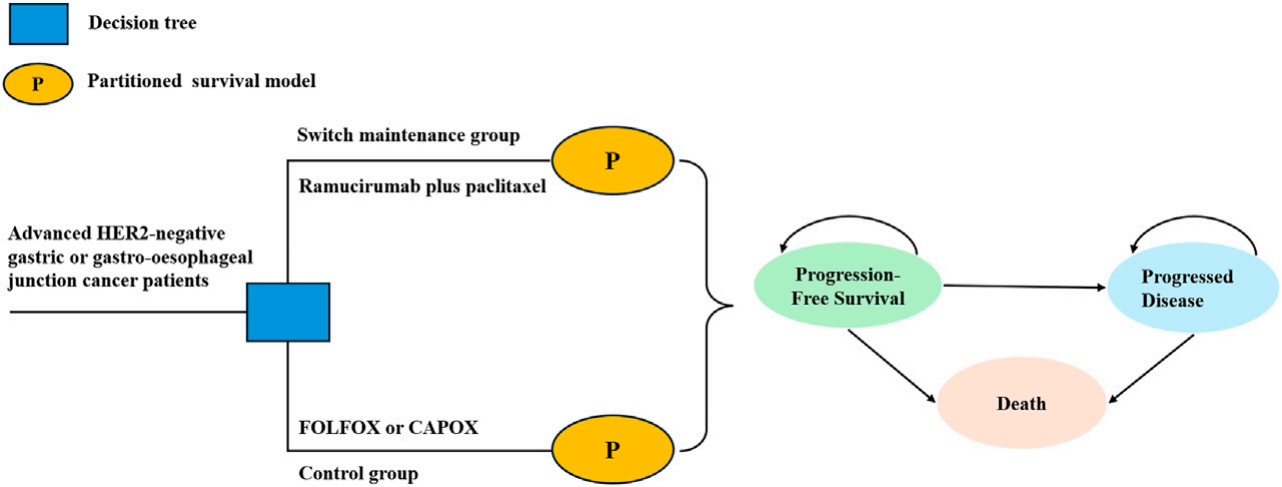
Structure of decision tree and partitioned survival model.

A partitioned survival model was developed by TreeAge Pro 2019. The model consisted of three mutually exclusive health states: PFS, progressive disease (PD), and death (Figure 1). All patients were assumed to begin in the PFS state and could transition to PD or death following treatment. Patient survival proportions were estimated using the area under the curve (AUC) of OS data from ARMANI, while PFS proportions were derived from PFS AUC. The PD proportion was calculated by subtracting the PFS proportion from the OS proportion. The model time horizon was set at 10 years, based on the assumption that more than 99% of patients would die within this period. The cycle length was 28 days, in alignment with the treatment regimen. The outcomes of the model were total cost, quality-adjusted life years (QALYs) and incremental cost-effectiveness ratio (ICER). The ICER was calculated to compare the cost-effectiveness of the switch maintenance group and control group. Combining the recommendations of the WHO-CHOICE guidelines (Bertram et al., 2021) and the China guidelines for pharmacoeconomic evaluations (Liu et al., 2020), the willingness-to-pay (WTP) threshold for China was set at $38,042.49 per QALY, which is three times the per capita GDP of China in 2023 (Central People’s Government of the People’s Republic of China, 2025). Consistent with other studies (Su et al., 2021; Chiang et al., 2021), $150,000 per QALY was used as the WTP threshold from the US perspective.

### Clinical data

Referring to the method of Guyot (Guyot et al., 2012), the WebPlotDigitizer program (version 4.6, https://automeris.io/WebPlotDigitizer/) was used to extract the survival data of OS and PFS from the Kaplan-Meier (KM) curves reported in the ARMANI study. The survHE, IPDfromKM, and flexsurv packages in R 4.4.2 were used to reconstruct the KM curves based on individual patient data (IPD). These data were used to fit various parametric survival models, including Exponential, Weibull, Gompertz, Log-logistic, Log-normal (Supplementary Table S2). Optimal models were selected by minimizing Akaike information criteria (AIC), Bayesian information criteria (BIC) and visual inspection (Table 1) (Williams et al., 2017). The fitting results are shown in Supplementary Figure S1–S10.

**TABLE 1:** Base-case model parameter.

Parameter	Baseline value	Range	Distribution	Reference
Log-logistic OS survival model of switch maintenance group	shape = 2.309, scale = 13.051	—	—	Randon et al. (2024)
Log-logistic OS survival model of control group	shape = 1.891, scale = 9.593	—	—	Randon et al. (2024)
Log-logistic PFS survival model of switch maintenance group	shape = 2.061, scale = 6.767	—	—	Randon et al. (2024)
Log-logistic PFS survival model of control group	shape = 1.697, scale = 3.579	—	—	Randon et al. (2024)
Log-logistic OS survival model of switch maintenance group in PD-L1-CPS≥5 patients	shape = 2.386, scale = 15.518	—	—	Randon et al. (2024)
Log-normal OS survival model of control group in PD-L1-CPS≥5 patients	meanlog = 2.376, sdlog = 0.805	—	—	Randon et al. (2024)
Log-normal PFS survival model of switch maintenance group in PD-L1-CPS≥5 patients	meanlog = 2.0989, sdlog = 0.7146	—	—	Randon et al. (2024)
Log-normal PFS survival model of control group in PD-L1-CPS≥5 patients	meanlog = 1.802, sdlog = 0.856	—	—	Randon et al. (2024)
Cost from the chinese perspective				
Enhanced CT per unit	23.693	10.659–106.647	Gamma	Qiu et al. (2023)
Blood biochemistry per unit	63.814	33.253–93.564	Gamma	Qiu et al. (2023)
Blood routine per unit	1.930	1.425–3.622	Gamma	Qiu et al. (2023)
Urine routine per unit	0.594	0.287–2.553	Gamma	Qiu et al. (2023)
Injection administration	0.802	0.158–0.643	Gamma	Qiu et al. (2023)
Intravenous injection	0.713	0.356–1.564	Gamma	Qiu et al. (2023)
Cost of terminal care	1,506.51	1,205.21–1,807.81	Gamma	Qiu et al. (2023)
Cost of BSC per cycle	267.78	214.22–321.34	Gamma	Zhou et al. (2025)
Ramucirumab/mg	5.23	4.186–6.279	Gamma	Shanghai sunshine procurement all. (2025)
Paclitaxel/mg	0.280	0.224–0.335	Gamma	Shanghai sunshine procurement all. (2025)
Ondansetron/mg	0.360	0.288–0.433	Gamma	Shanghai sunshine procurement all. (2025)
Dexamethasone/mg	0.018	0.0148–0.0221	Gamma	Shanghai sunshine procurement all. (2025)
Diphenhydramine/mg	0.103	0.0823–0.123	Gamma	Shanghai sunshine procurement all. (2025)
Capecitabine/mg	0.000653	0.000522–0.000783	Gamma	Shanghai sunshine procurement all. (2025)
Calcium folinate/mg	0.0355	0.0284–0.0426	Gamma	Shanghai sunshine procurement all. (2025)
Oxaliplatin/mg	0.881	0.705–1.058	Gamma	Shanghai sunshine procurement all. (2025)
5-Fluorouracil/mg	0.0183	0.0146–0.0220	Gamma	Shanghai sunshine procurement all. (2025)
Irinotecan/mg	0.0955	0.0764–0.115	Gamma	Shanghai sunshine procurement all. (2025)
Cost from the US perspective				
Enhanced CT per unit	530.79	424.63–636.95	Gamma	Centers for Medicare and Medicaid Services. (2025)
Blood biochemistry per unit	10.56	8.45–12.67	Gamma	Centers for Medicare and Medicaid Services. (2025)
Blood routine per unit	7.77	6.22–9.32	Gamma	Centers for Medicare and Medicaid Services. (2025)
Urine routine per unit	3.17	2.54–3.80	Gamma	Centers for Medicare and Medicaid Services. (2025)
Injection administration	13.91	11.13–16.69	Gamma	Centers for Medicare and Medicaid Services. (2025)
Intravenous injection	223.30	178.64–267.96	Gamma	Centers for Medicare and Medicaid Services. (2025)
Cost of terminal care	19,247.03	15,397.62–23,096.44	Gamma	Zhu et al. (2023)
Cost of BSC per cycle	2,262.59	1,810.07–2,715.11	Gamma	Zhu et al. (2023)
Ramucirumab/mg	15	12–18	Gamma	IBM Micromedex IBM Corporation. (2025)
Paclitaxel/mg	0.2882	0.23056–0.34584	Gamma	IBM Micromedex IBM Corporation. (2025)
Ondansetron/mg	0.1056	0.08448–0.12672	Gamma	IBM Micromedex IBM Corporation. (2025)
Dexamethasone/mg	0.02255	0.01804–0.02706	Gamma	IBM Micromedex IBM Corporation. (2025)
Diphenhydramine/mg	0.0602	0.04816–0.07224	Gamma	IBM Micromedex IBM Corporation. (2025)
Capecitabine/mg	0.002	0.0016–0.0024	Gamma	IBM Micromedex IBM Corporation. (2025)
Calcium folinate/mg	0.07026	0.056208–0.084312	Gamma	IBM Micromedex IBM Corporation. (2025)
Oxaliplatin/mg	0.6	0.48–0.72	Gamma	IBM Micromedex IBM Corporation. (2025)
5-Fluorouracil/mg	0.01027	0.008216–0.012324	Gamma	IBM Micromedex IBM Corporation. (2025)
Irinotecan/mg	0.3	0.24–0.36	Gamma	IBM Micromedex IBM Corporation. (2025)
AEs management costs from Chinese perspective				
Neutropenia	136.792	109.418–164.147	Gamma	Qiu et al. (2023)
Peripheral neuropathy	1,097.008	8,77.607–1,316.410	Gamma	Pike et al. (2012)
Hypertension	17.824	14.261–21.387	Gamma	Qiu et al. (2023)
AEs management costs from US perspective				
Neutropenia	18,360.82	14,688.66–22,032.98	Gamma	Stenehjem et al. (2023)
Peripheral neuropathy	14,184.2	11,347.36–17,021.04	Gamma	Stenehjem et al. (2023)
Hypertension	19,272.01	15,417.61–23,126.41	Gamma	Zhang et al. (2021)
Utility				
PFS	0.797	0.64–0.96	Beta	Tsuchiya et al. (2002)
PD	0.577	0.46–0.69	Beta	Holden et al. (2011)
Disutility				
Neutropenia	0.2	0.16–0.24	Beta	Nafees et al. (2017)
Peripheral neuropathy	0.16	0.128–0.192	Beta	Swinburn et al. (2015)
Hypertension	0.04	0.032–0.048	Beta	Nafees et al. (2017)
Risk of serious AEs in switch maintenance group				
Neutropenia (%)	26.24	20.99–31.49	Beta	Randon et al. (2024)
Peripheral neuropathy (%)	6	4.80–7.20	Beta	Randon et al. (2024)
Hypertension (%)	6	4.80–7.20	Beta	Randon et al. (2024)
Risk of serious AEs in control group				
Neutropenia (%)	9.63	7.70–11.56	Beta	Randon et al. (2024)
Peripheral neuropathy (%)	7	5.60–8.40	Beta	Randon et al. (2024)
Others				
Weight of Chinese patients (kg)	69	55.2–82.88	Gamma	Central People’s Government of the People’s Republic of China. (2021)
Weight of US patients (kg)	75	60–90	Gamma	Luo et al. (2024)
BSA of Chinese patients (m^2^)	1.74	1.392–2.088	Gamma	Central People’s Government of the People’s Republic of China (2021)
BSA of US patients (m^2^)	1.8	1.44–2.16	Gamma	Li et al. (2021)
Discount rate in China (%)	5	0–8	Fixed	Liu et al. (2020)
Discount rate in US (%)	3	0–8	Fixed	Sanders et al. (2016)

Abbreviations: OS, overall survival; PFS, progression-free survival; CT, computed tomography; AE, adverse event; PD, progressed disease; BSC, best supportive care; US, united states; PD-L1, programmed cell death ligand 1; CPS, combined positive score.

### Costs and utilities

This study was conducted from the perspective of the Chinese and US healthcare system, considering only direct medical costs, including drug costs, drug injections costs, examination costs, management costs of each adverse events (AEs), BSC cost, and terminal care costs. We assumed that AEs occurred exclusively during the first model cycle (Su et al., 2021). Given that grade <3 AEs generally have minimal clinical impact and are managed either expectantly or with supportive care, only grade ≥3 AEs with an incidence >5% were incorporated into the model to maintain methodological simplicity. The included AEs encompassed peripheral neuropathy, hypertension, and neutropenia. The cost of drugs was obtained from the Shanghai Sunshine pharmaceutical procurement network (Shanghai sunshine procurement all, 2025) and Red Book^®^ (IBM Micromedex IBM Corporation, 2025), and the cost of examinations, terminal care, drug injections and management costs of each AE were obtained from Medicare Physician Fee Schedule and published studies (Qiu et al., 2023; Tang et al., 2022; Centers for Medicare and Medicaid Services, 2025). Given price variations across multiple manufacturers, the median price was applied as the drug cost parameter. All Chinese costs were adjusted to 2023 values using the China Consumer Price Index (CPI) and are presented in USD ($1 = ¥7.0467). All US cost were adjusted to 2023 dollars using Tom’s Inflation Calculator (Medical-care inflation, 2025). Waste of medication was not considered in our study. The detailed cost data are shown in Table 1.

Health utility values for PFS and PD states were derived from published studies, with values set at 0.797 for PFS from TOGA trial (Tsuchiya et al., 2002), 0.577 for PD from National Institute for Health and Clinical Excellence (NICE) (Holden et al., 2011), and a utility of 0 for death. The disutility of AEs was also incorporated into the model. The disutility values for peripheral neuropathy, neutropenia, and hypertension were 0.16, 0.2, and 0.04, respectively, as reported in published studies (Swinburn et al., 2015; Nafees et al., 2017). All costs and utilities were discounted at an annual rate of 5% for the Chinese perspective and 3% for the US perspective (Liu et al., 2020; Sanders et al., 2016).

### Sensitivity analysis

Both one-way sensitivity analysis and probabilistic sensitivity analysis (PSA) were conducted to assess the robustness of the results. One-way sensitivity analysis evaluated the impact of individual parameters on model outcomes by varying each parameter within a range of ±20% from its baseline value while keeping all other variables constant. Tornado diagram was used to present the results of one-way sensitivity analysis. For the discount rate, we followed the recommendations of the China guidelines for pharmacoeconomic evaluations and varied it between 0% 8% (Liu et al., 2020). PSA was performed using a Monte Carlo simulation with 5,000 iterations, where each parameter was randomly sampled from predefined distributions. Results from the PSA were represented by an incremental cost-effectiveness scatter plot and a cost-effectiveness acceptability curve (CEAC), illustrating the probability of the treatment being cost-effectiveness at different WTP thresholds. Gamma distributions were assumed for cost, weight, and body surface area, while beta distributions were used for utility and probability parameters.

### Scenarios analysis

To further assess the cost-effectiveness of ramucirumab, we employed the quartile range method to analyze various pricing scenarios where the price of ramucirumab was reduced to 75%, 50%, and 25% of its original price. This analysis aimed to observe the impact on the ICER and the probability of cost-effectiveness acceptability.

### Subgroup analysis

Programmed cell death ligand 1 (PD-L1) may act on VEGFR2 to promote cancer cell angiogenesis and metastasis (Choi et al., 2023; Yang et al., 2021). Therefore, the expression of PD-L1 combined positive score (CPS) significantly impacts the efficacy of ramucirumab. Based on this rationale, we conducted a subgroup analysis in patients with PD-L1 CPS ≥5.

## Results

### Base-case analysis and subgroup analysis

The base-case analysis results are presented in Table 2. In the overall population from the Chinese perspective, the switch maintenance group provided an additional 0.15 QALYs compared to the control group, but incurred incremental costs of $56,738.52, yielding an ICER of $373,219.84 per QALY. For the PD-L1 CPS ≥5 subgroup, the switch maintenance group provided an additional 0.24 QALYs with incremental costs of $62,741.24, resulting in an ICER of $266,259.94 per QALY. In both the overall population and PD-L1 CPS ≥5 subgroup, the ICERs substantially exceeded China’s current WTP threshold ($38,042.49 per QALY).

**TABLE 2:** The cost and outcome results of the base-case analysis.

Parameters	Overall population				PD–L1 CPS ⩾5 population			
	Chinese perspective		United States perspective		Chinese perspective		United States perspective	
	Switch maintenance group	Control group	Switch maintenance group	Control group	Switch maintenance group	Control group	Switch maintenance group	Control group
Total cost ($)	69,852.42	13,114.09	256,611.11	713,60.56	75,556.47	12,815.23	276,370.43	70,263.30
Incremental costs ($)	56,738.52	–	185,250.55	–	62,741.24	–	206,107.13	–
Overall QALYs	0.89	0.74	0.91	0.76	1.02	0.79	1.05	0.80
Incremental QALYs	0.15	–	0.16	–	0.24	–	0.25	–
ICER ($/QALY)	**373,219.84**		**1,193,220.74**		**266,259.94**		**835,740.90**	

Abbreviations: CPS, combined positive score; ICER, incremental cost-effectiveness ratio; QALYs, quality-adjusted life-years.

The bold font represents the ratio of incremental costs to incremental QALYs between the two treatment groups.

From the US perspective, the switch maintenance group provided an additional 0.16 QALYs in the overall population versus control, with incremental costs of $185,250.55, yielding an ICER of $1,193,220.74 per QALY. In the PD-L1 CPS ≥5 subgroup, it provided 0.25 additional QALYs at incremental costs of $206,107.13, resulting in an ICER of $835,740.90 per QALY. All ICERs significantly exceeded the US WTP threshold ($150,000.00 per QALY) across both overall populations and subgroup populations.

### Sensitivity analysis

**FIGURE 2:**
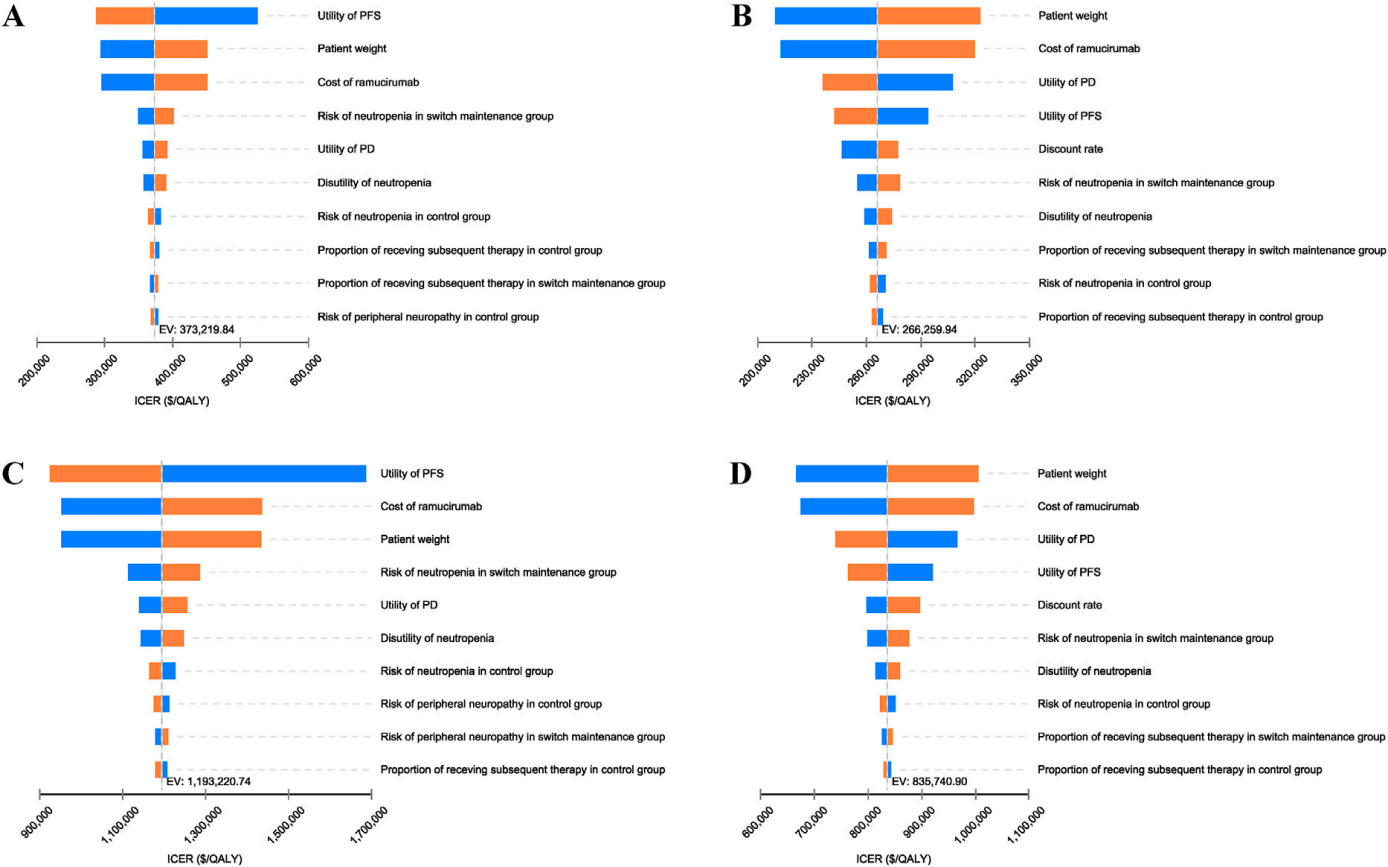
Tornado diagram of one-way sensitivity analysis of switch maintenance group versus control group. **(A)** Overall population from Chinese perspective, **(B)** PD-L1 CPS ⩾5 population from Chinese perspective, **(C)** Overall population from United States perspective, **(D)** PD-L1 CPS ⩾5 population from United States perspective. ICER, incremental cost-effectiveness ratio; PD, progressive disease; PFS, Progression-free survival.

The results of the tornado diagram are shown in Figure 2. In the overall population, the top three influential parameters of the ICER were the utility of PFS, the price of ramucirumab, and patient weight. In the PD-L1 CPS ≥5 population, the top three influential parameters were body weight, the price of ramucirumab, and the utility of PD. Other parameters, such as the discount rate, incidence of AEs, and disutility values, had slight impacts on the ICER. These hierarchical patterns remained consistent in both Chinese and US perspectives. However, no single parameter factors could reduce the ICER below current WTP threshold in China and US. This is consistent with our results in base-case analysis.

**FIGURE 3:**
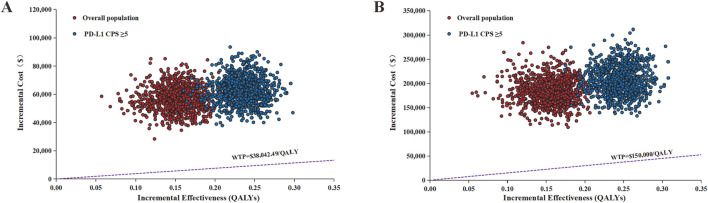
Incremental cost-effectiveness scatter plot of switch maintenance group versus control group. **(A)** Chinese perspective **(B)** United States perspective. QALYs, quality-adjusted life years; WTP, willingness-to-pay.

**FIGURE 4:**
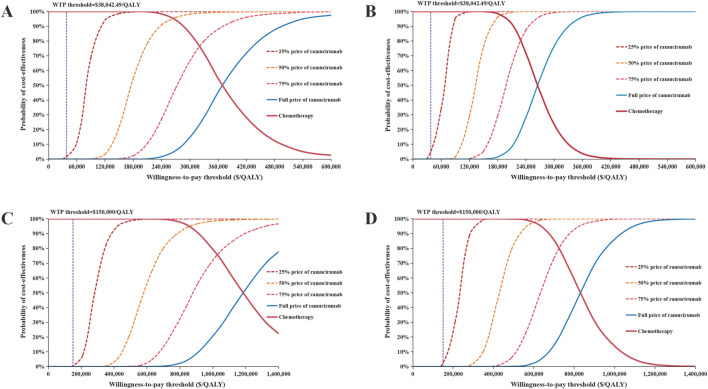
Cost-effectiveness acceptability curve of different ramucirumab prices. **(A)** Overall population from Chinese perspective, **(B)** PD-L1 CPS ⩾5 population from Chinese perspective, **(C)** Overall population from United States perspective, **(D)** PD-L1 CPS ⩾5 population from United States perspective. Abbreviations: QALYs, quality-adjusted life years; WTP, willingness-to-.pay.

The results of the PSA are shown in Figures 3, 4. The scatter plots demonstrated that none of the 5,000 Monte Carlo iterations yielded ICERs below current WTP thresholds in either China or US perspectives—consistent across both the overall population and PD-L1 CPS ≥5 subgroup. Furthermore, the CEAC demonstrated that the acceptable probability of full-price ramucirumab-based switch maintenance regimen was 0% in both China and the US in the overall population and the PD-L1 CPS ≥5 subgroup.

### Scenario analysis

The results of the scenario analysis are shown in Figure 4 and Table 3. The CEACs demonstrated that at current WTP thresholds in both China and the US, the switch maintenance therapy showed no cost-effectiveness advantage over the control group even with a 75% reduction in ramucirumab price. Further analysis revealed that when the cost of ramucirumab dropped to $0.743/mg (14.2% of the current price) in China and $2.088/mg (13.92% of the current price) in the US for the overall population, the probability of ramucirumab being cost-effective will increase to 50%.

**TABLE 3:** Results of scenarios analysis of ramucirumab at different prices.

Parameter	Perspective	Ramucirumab price/mg ($)	Total costs ($)	QALYs	ICER($/QALY)	Cost-effectiveness probability (%)
Overall population						
100% price of ramucirumab	China	5.2322	69,852.42	0.89	373,219.84	0
	United States	15	256,611.11	0.91	1,193,220.74	0
75% price of ramucirumab	China	3.9242	55,044.13	0.89	275,812.24	0
	United States	11.25	209,632.44	0.91	890,625.58	0
50% price of ramucirumab	China	2.6161	40,234.72	0.89	178,397.19	0
	United States	7.5	162,653.77	0.91	588,030.42	0
25% price of ramucirumab	China	1.3081	25,426.44	0.89	80,989.59	0
	United States	3.75	115,675.11	0.91	285,435.26	0
PD-L1 CPS ⩾5 population						
100% price of ramucirumab	China	5.2322	75,556.47	1.02	266,259.94	0
	United States	15	276,370.43	1.05	835,740.90	0
75% price of ramucirumab	China	3.9242	59,820.01	1.02	199,477.88	0
	United States	11.25	226,670.71	1.05	634,214.22	0
50% price of ramucirumab	China	2.6161	44,082.34	1.02	132,690.71	0
	United States	7.5	176,971.00	1.05	432,687.54	0
25% price of ramucirumab	China	1.3081	28,345.88	1.02	65,908.65	0
	United States	3.75	127,271.28	1.05	231,160.86	0

Abbreviations: ICER, incremental cost-effectiveness ratio; QALY, quality-adjusted life years; US, United State.

## Discussion

For patients with HER2-negative advanced GC or GEJC, fluoropyrimidine and platinum-based chemotherapy remains the first-line treatment (National Comprehensive Cancer Network, 2024; Lordick et al., 2022). However, the OS with first-line treatment rarely exceeds 12 months, and only 40%–50% of patients in clinical trials qualify for subsequent anticancer therapies (Janjigian et al., 2024), highlighting an urgent need to optimize upfront treatment strategies. The ARMANI trial offers a promising therapeutic approach for this population. However, the high cost of ramucirumab poses a serious challenge to patient accessibility, and a thorough evaluation of the economic feasibility of this regimen is needed.

The analysis results underscore that, under current WTP threshold in China and US, the ramucirumab plus paclitaxel regimen lacks cost-effectiveness compared to continuing oxaliplatin-based chemotherapy in HER2-negative advanced GC or GEJC patients—a conclusion consistent across both the overall population and the PD-L1 CPS ≥5 population. The one-way sensitivity analysis revealed that utility of PFS and PD, patient weight, and the price of ramucirumab significantly influenced model outcomes. However, no single parameter adjustment could reduce the ICER below current WTP threshold in China or US, a finding consistent across both the overall population and the PD-L1 CPS ≥5 population, thereby validating the robustness of our analysis.

Our findings align with existing cost-effectiveness analyses of the regimen of ramucirumab plus paclitaxel. Li and his colleagues evaluated ramucirumab plus paclitaxel versus placebo plus paclitaxel as a second-line therapy for advanced GC or GEJC within Chinese healthcare system (Li et al., 2020). Their study concluded that when the price of ramucirumab exceeded $1.6667 per mg, the probability of the ICER surpassing WTP threshold in China ($26,021.9 per QALY) was 100%. A Japanese study reported an ICER of $2,417,664.31/QALY for ramucirumab plus paclitaxel versus paclitaxel monotherapy, far exceeding WTP threshold in Japan of $674,536.26 per QALY (Saito et al., 2017). Additionally, NICE concluded that ramucirumab plus paclitaxel lacks cost-effectiveness for advanced GC after prior chemotherapy (Büyükkaramikli et al., 2017). The lacks of cost-effectiveness of the ramucirumab plus paclitaxel regimen may be primarily attributed to the sustained administration of ramucirumab, which substantially elevates treatment costs. In the aforementioned studies, *ramucirumab* was administered at 8 mg/kg on days 1 and 8 of each 28-day cycle, with no predefined treatment duration limit.

Scenario analysis further sheds light on the economic toxicity of ramucirumab under real-world pricing policies. Ramucirumab was approved for marketing by National Medical Products Administration in 2022, but it has not been included in China’s National Reimbursement Drug List (NRDL), which increases the financial pressure on patients. Nevertheless, our results suggest that even if ramucirumab were included in the NRDL (with an average price reduction of 63% after negotiation in 2024 according to the National Healthcare Security Administration (China’s National Healthcare Security Administration, 2024)), ramucirumab plus paclitaxel would still not be cost-effective. To achieve cost-effectiveness, ramucirumab would require price reductions to 14.2% of full price ($0.743/mg) in China. As a high-burden country for GC, China reported approximately 509,000 new GC cases and 400,000 deaths in 2022 (Xia et al., 2022)—the highest global incidence—necessitating policymakers to implement aggressive strategies to alleviate patient financial toxicity and broaden access to this promising therapy. Furthermore, China is currently the second-largest pharmaceutical market globally and dominates the GC treatment space in the Asia-Pacific region, with a revenue share of 27.1% in 2024 (Grand view research, 2025). The manufacturer must reduce the price of ramucirumab or optimize its dosing regimen, for example, by exploring the feasibility of reducing the dose and shortening the duration of treatment, to increase the economic feasibility of ramucirumab and cover more patients who can benefit from it.

Despite declining incidence and mortality of GC in the US, its substantial treatment costs continue to impose significant burdens on the US healthcare system (Thrift and El-Serag, 2020). This economic burden largely stems from exorbitant drug pricing—US pharmaceutical costs in 2022 averaged nearly threefold higher than those in other OECD nations (Mulcahy et al., 2024). Such pricing threatens patient’s access to quality hospital care, making healthcare affordability and accessibility top priorities for most Americans (American Hospital Association, 2024). On 12 May 2025, the White House issued the Executive Order “Most Favored Nation Pricing for Prescription Drugs Delivered to American Patients,” projected to reduce US drug prices by 59% (The White House, 2025). Nevertheless, our analysis demonstrates that even with a 75% price reduction for ramucirumab, the ramucirumab plus paclitaxel remains lacks cost-ineffectiveness in the US perspective. Consequently, alongside advocating for policy interventions, future clinical trials should prioritize dose optimization and alternative administration strategies. The positive effects of proving that lower doses or less intensive schedules retain therapeutic activity include reduced toxicity and large price reductions, leading to better cost-effectiveness and greater access to treatments that improve survival (Tannock et al., 2025).

Our study has some limitations. First, drug prices from a Chinese perspective were sourced from the Shanghai Sunshine Pharmaceutical Procurement Network. Given the regional variations in drug pricing across different areas of China, the ICER values in this study may not be directly generalizable to other Chinese regions. Nevertheless, the large differences between ICER and WTP thresholds suggest that the conclusions drawn from our study remain robust across different regions. Second, manually extracting data points from the KM curves reported in the ARMANI trial using the WebPlotDigitizer for curve reconstruction could lead to uncontrolled methodological biases. Third, since the ARMANI trial enrolled a predominantly Caucasian population, extrapolating its findings to assess the cost-effectiveness of switch maintenance therapy in China may introduce uncertainty due to inherent heterogeneity between the study populations. Fourth, as the ARMANI trial did not specify post-progression treatment regimens or their proportions, we assumed intravenous irinotecan as the standard subsequent therapy for all patients based on NCCN and CSCO guidelines. However, real-world treatment pathways are often more complex and heterogeneous. Despite the limitations of our study, the finding that the ICER of the ramucirumab plus paclitaxel regimen is tenfold higher than current WTP threshold in China and US suggests that these limitations are unlikely to reverse the conclusions. Thus, our results still offer critical economic insights for policymakers, pharmaceutical manufacturers, and healthcare providers.

This study highlights several key directions for further investigation. First, future research should focus on identifying patient subgroups that may benefit cost-effectively from switch maintenance therapy through the exploration of additional tumor biomarkers such as CLDN18.2 or mismatch repair status. Second, alternative ramucirumab dosing strategies warrant exploration, including reduced-dose regimens or treatment duration limitations (Tannock et al., 2025). Finally, comparative cost-effectiveness analyses of switch maintenance therapy should be conducted across diverse healthcare systems and socioeconomic contexts to assess generalizability.

## Conclusion

In summary, for Chinese and US patients with previously treated advanced HER2-negative GC or GEJC—whether in the overall population or the PD-L1 CPS ⩾5 population—the ramucirumab plus paclitaxel regimen lacks cost-effectiveness as first-line switch maintenance therapy compared to continuing oxaliplatin-based chemotherapy. Ramucirumab would need to reduce its price to 14.2% of the original price ($0.743/mg) in China and 13.92% of the original price ($2.088/mg) in the US, to have a 50% probability of being cost-effective.

## Data Availability

The original contributions presented in the study are included in the article/Supplementary Material, further inquiries can be directed to the corresponding authors.
