# Supplementary material for: Ramucirumab plus paclitaxel as switch maintenance in patients with advanced HER2-negative gastric or gastro-oesophageal junction cancer: a cost-effectiveness analysis
Source: Front Pharmacol. 2025 Sep 12;16:1616826. doi: 10.3389/fphar.2025.1616826 (PMC12463587; doi:10.3389/fphar.2025.1616826)
Supplement: Supplementary file 1 [file DataSheet1.pdf]

Supplementary Material

Supplementary Table S1. CHEERS Checklist 2022.

Supplementary Table S2. Summary of the statistical goodness-of-fit of Kaplan-Meier survival curves.

Supplementary Figure S1. Kaplan-Meier curves of the reconstructed overall population. A: Overall survival curve; B: Progression-free survival curve.

Supplementary Figure S2. Kaplan-Meier curves of the reconstructed PD-L1 CPS ⩾ 5 patient population. A: Overall survival curve; B: Progression-free survival curve.

Supplementary Figure S3. Extrapolation of the OS curves for the overall population to the switch maintenance group.

Supplementary Figure S4. Extrapolation of the OS curves for the overall population to the control group.

Supplementary Figure S5. Extrapolation of the PFS curves for the overall population to the switch maintenance group.

Supplementary Figure S6. Extrapolation of the PFS curves for the overall population to the control group.

Supplementary Figure S7. Extrapolation of the OS curves for the PDL1-CPS⩾5 population to the switch maintenance group.

Supplementary Figure S8. Extrapolation of the OS curves for the PDL1-CPS⩾5 population to the control group.

Supplementary Figure S9. Extrapolation of the PFS curves for the PDL1-CPS⩾5 population to the switch maintenance group.

Supplementary Figure S10. Extrapolation of the PFS curves for the PDL1-CPS⩾5 population to the switch maintenance group.

1 Supplementary Tables

Supplementary Table S1. CHEERS Checklist 2022.

	Item	Guidance for Reporting	Reported
**TITLE**			
Title	1	Identify the study as an economic evaluation and specify the interventions being compared.	Yes
**ABSTRACT**			
Abstract	2	Provide a structured summary that highlights context, key methods, results and alternative analyses.	Yes
**INTRODUCTION**			
Background and objectives	3	Give the context for the study, the study question and its practical relevance for decision making in policy or practice.	Yes
**METHODS**			
Health economic analysis plan	4	Indicate whether a health economic analysis plan was developed and where available.	Not applicable
Study population	5	Describe characteristics of the study population (such as age range, demographics, socioeconomic, or clinical characteristics).	Yes
Setting and location	6	Provide relevant contextual information that may influence findings.	Yes
Comparators	7	Describe the interventions or strategies being compared and why chosen.	Yes
Perspective	8	State the perspective(s) adopted by the study and why chosen.	Yes
Time horizon	9	State the time horizon for the study and why appropriate.	Yes
Discount rate	10	Report the discount rate(s) and reason chosen.	Yes
Selection of outcomes	11	Describe what outcomes were used as the measure(s) of benefit(s) and harm(s).	Yes
Measurement of outcomes	12	Describe how outcomes used to capture benefit(s) and harm(s) were measured.	Yes
Valuation of outcomes	13	Describe the population and methods used to measure and value outcomes.	Yes
Measurement and valuation of resources and costs	14	Describe how costs were valued.	Yes
Currency, price date, and conversion	15	Report the dates of the estimated resource quantities and unit costs, plus the currency and year of conversion.	Yes
Rationale and description of model	16	If modelling is used, describe in detail and why used. Report if the model is publicly available and where it can be accessed.	Yes

Analytics and assumptions	17	Describe any methods for analysing or statistically transforming data, any extrapolation methods, and approaches for validating any model used.	Yes
Characterizing heterogeneity	18	Describe any methods used for estimating how the results of the study vary for sub-groups.	Yes
Characterizing distributional effects	19	Describe how impacts are distributed across different individuals or adjustments made to reflect priority populations.	Not applicable
Characterizing uncertainty	20	Describe methods to characterize any sources of uncertainty in the analysis.	Yes
Approach to engagement with patients and others affected by the study	21	Describe any approaches to engage patients or service recipients, the general public, communities, or stakeholders (e.g., clinicians or payers) in the design of the study.	Not applicable
**RESULTS**			
Study parameters	22	Report all analytic inputs (e.g., values, ranges, references) including uncertainty or distributional assumptions.	Yes
Summary of main results	23	Report the mean values for the main categories of costs and outcomes of interest and summarise them in the most appropriate overall measure.	Yes
Effect of uncertainty	24	Describe how uncertainty about analytic judgments, inputs, or projections affect findings. Report the effect of choice of discount rate and time horizon, if applicable.	Yes
Effect of engagement with patients and others affected by the study	25	Report on any difference patient/service recipient, general public, community, or stakeholder involvement made to the approach or findings of the study	Not applicable
**DISCUSSION**			
Study findings, limitations, generalizability, and current knowledge	26	Report key findings, limitations, ethical or equity considerations not captured, and how these could impact patients, policy, or practice.	Yes
**OTHER RELEVANT INFORMATION**			
Source of funding	27	Describe how the study was funded and any role of the funder in the identification, design, conduct, and reporting of the analysis	Yes
Conflicts of interest	28	Report authors conflicts of interest according to journal or International Committee of Medical Journal Editors requirements.	Yes

Husereau D, Drummond M, Augustovski F, de Bekker-Grob E, Briggs AH, Carswell C, Caulley L, Chaiyakunapruk N, Greenberg D, Loder E, Mauskopf J, Mullins CD, Petrou S, Pwu RF, Staniszewska S; CHEERS 2022 ISPOR Good Research Practices Task Force. Consolidated Health Economic Evaluation Reporting Standards 2022 (CHEERS 2022) Statement: Updated Reporting Guidance for Health Economic Evaluations. BMJ. 2022;376:e067975.

Supplementary Table S2. Summary of the statistical goodness-of-fit of Kaplan-Meier survival curves.

Distribution	Switch maintenance group, AIC	Switch maintenance group, BIC	Control group, AIC	Control group, BIC
OS for overall population				
Exponential	950.8476	953.8175	835.3408	838.2535
Weibull	925.3062	931.2459	825.7458	831.5711
Gompertz	942.5279	948.4675	833.6266	839.4520
Log-logistic	**913.1722**	**919.1119**	**824.4644**	**830.2897**
Log-normal	918.5555	924.4952	824.7552	830.5805
PFS for overall population				
Exponential	833.7583	836.7282	708.9919	711.9046
Weibull	821.7046	827.6442	710.9851	716.8104
Gompertz	834.0017	839.9414	706.2947	712.1200
Log-logistic	**804.7008**	**810.6404**	**690.0986**	**695.9239**
Log-normal	804.9032	810.8428	711.3944	717.2197
OS for PDL1-CPS⩾5 patients				
Exponential	210.2530	212.0142	181.5072	183.0336
Weibull	202.9122	206.4346	179.5465	182.5993
Gompertz	207.4228	210.9452	182.6578	185.7105
Log-logistic	**200.3665**	**203.8889**	175.5855	178.6382
Log-normal	200.8396	204.3620	**174.5860**	**177.6387**
PFS for PDL1-CPS⩾5 patients				
Exponential	214.5111	216.2723	108.3769	109.9033
Weibull	209.1121	212.6345	109.7397	112.7925
Gompertz	214.8418	218.3642	109.6199	112.6727
Log-logistic	202.1556	205.6780	101.2599	104.3126
Log-normal	**201.3514**	**204.8738**	**100.9605**	**104.0132**

2 Supplementary Figures

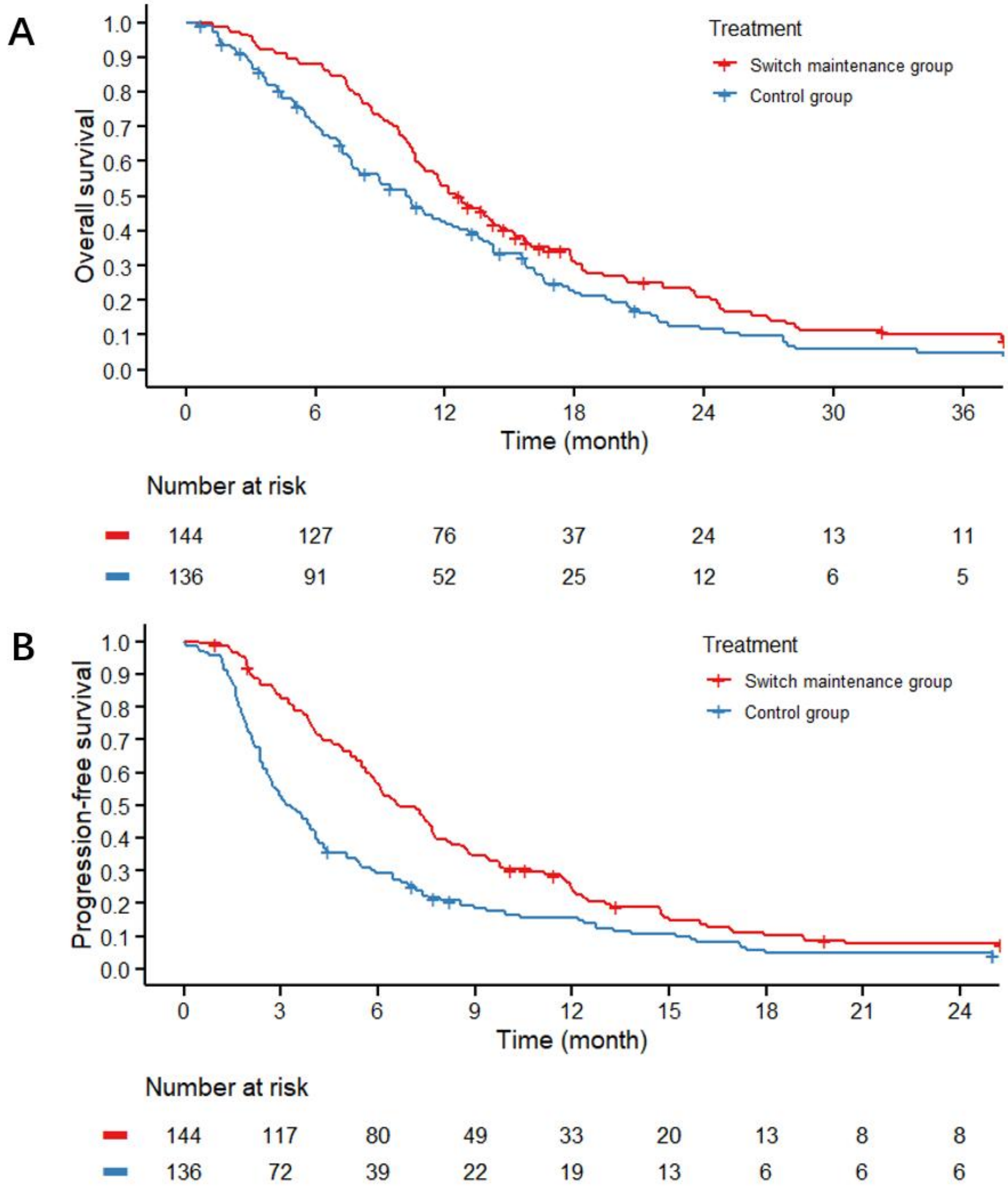

Supplementary Figure S1. Kaplan-Meier curves of the reconstructed overall population. A: Overall survival curve; B: Progression-free survival curve.

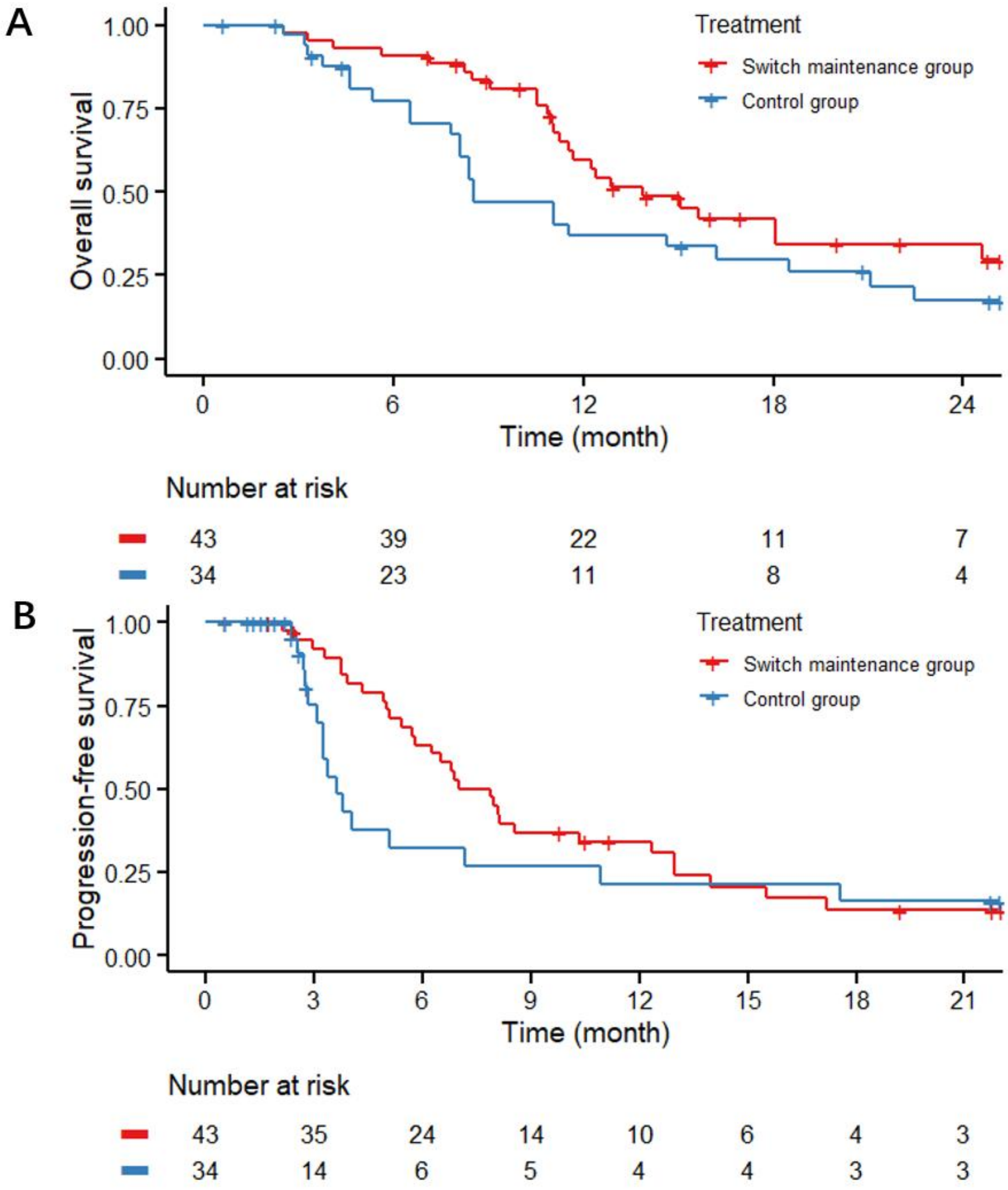

Supplementary Figure S2. Kaplan-Meier curves of the reconstructed PD-L1 CPS ⩾ 5 patient population. A: Overall survival curve; B: Progression-free survival curve.

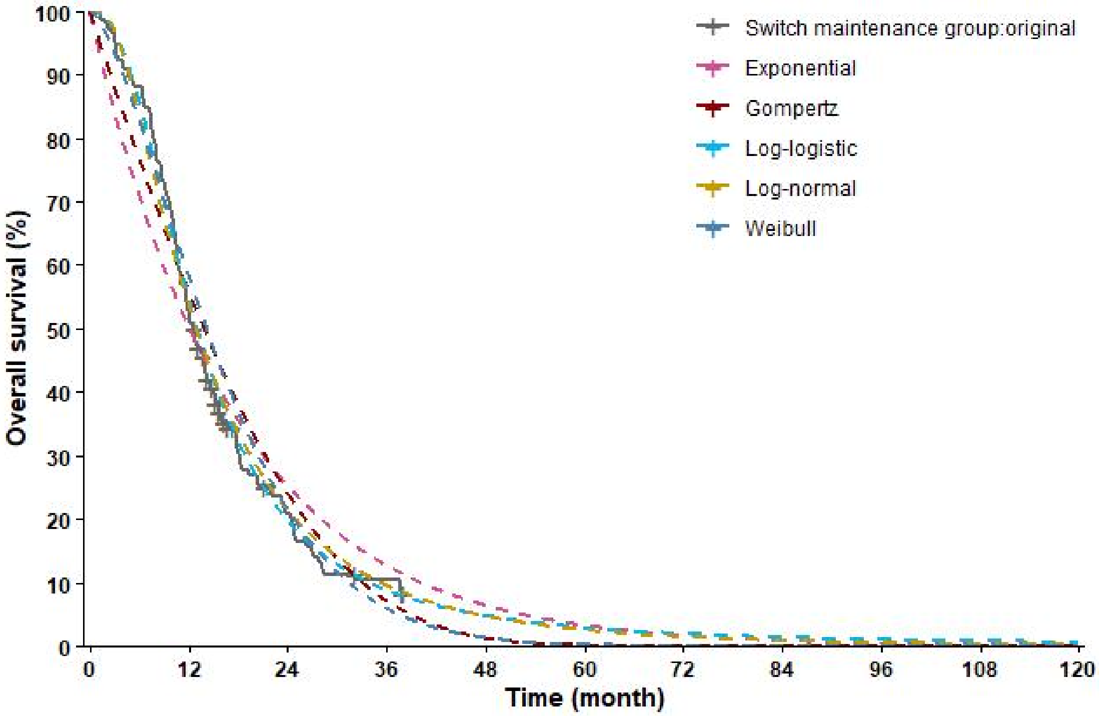

Supplementary Figure S3. Extrapolation of the OS curves for the overall population to the switch maintenance group.

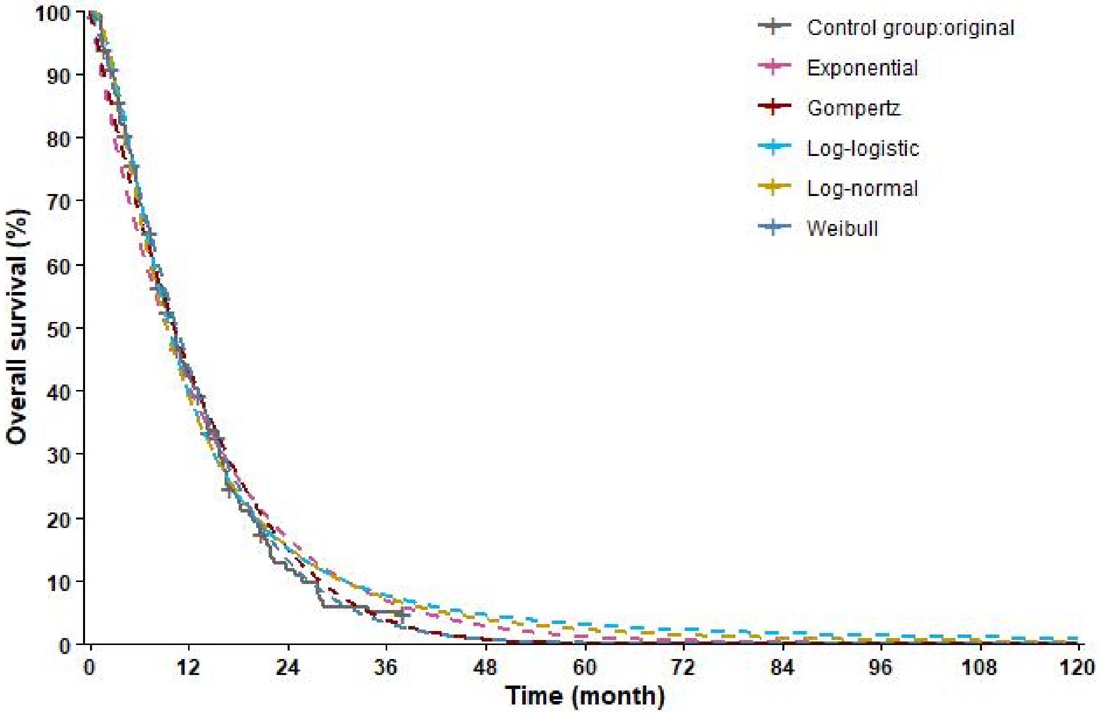

Supplementary Figure S4. Extrapolation of the OS curves for the overall population to the control group.

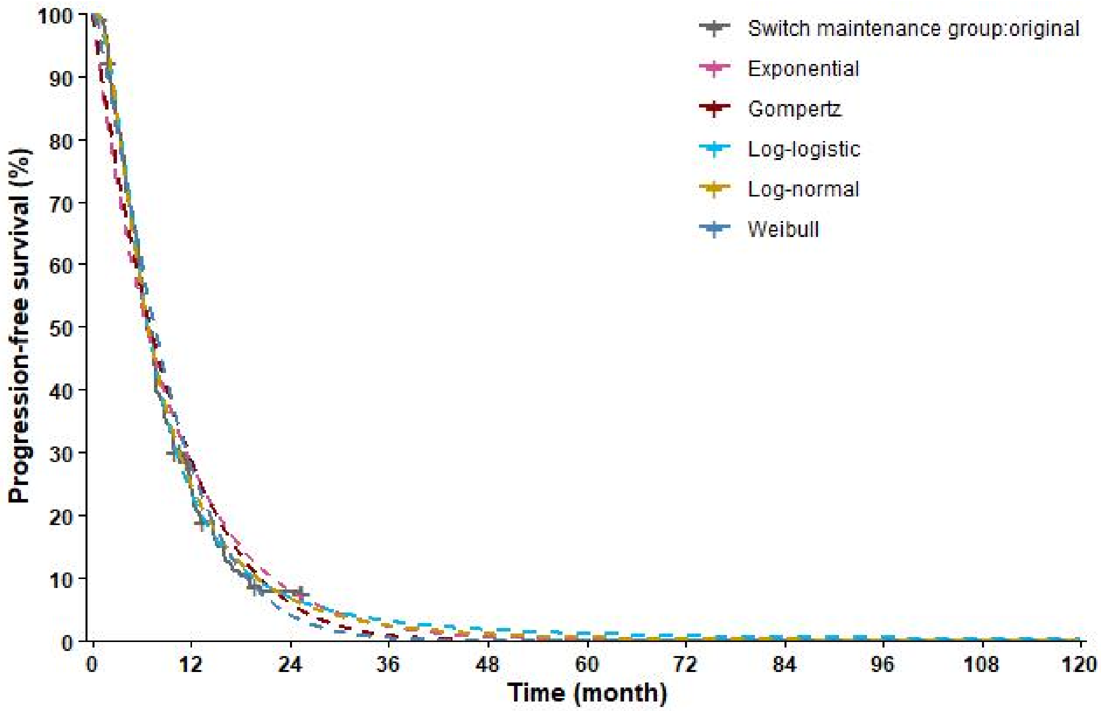

Supplementary Figure S5. Extrapolation of the PFS curves for the overall population to the switch maintenance group.

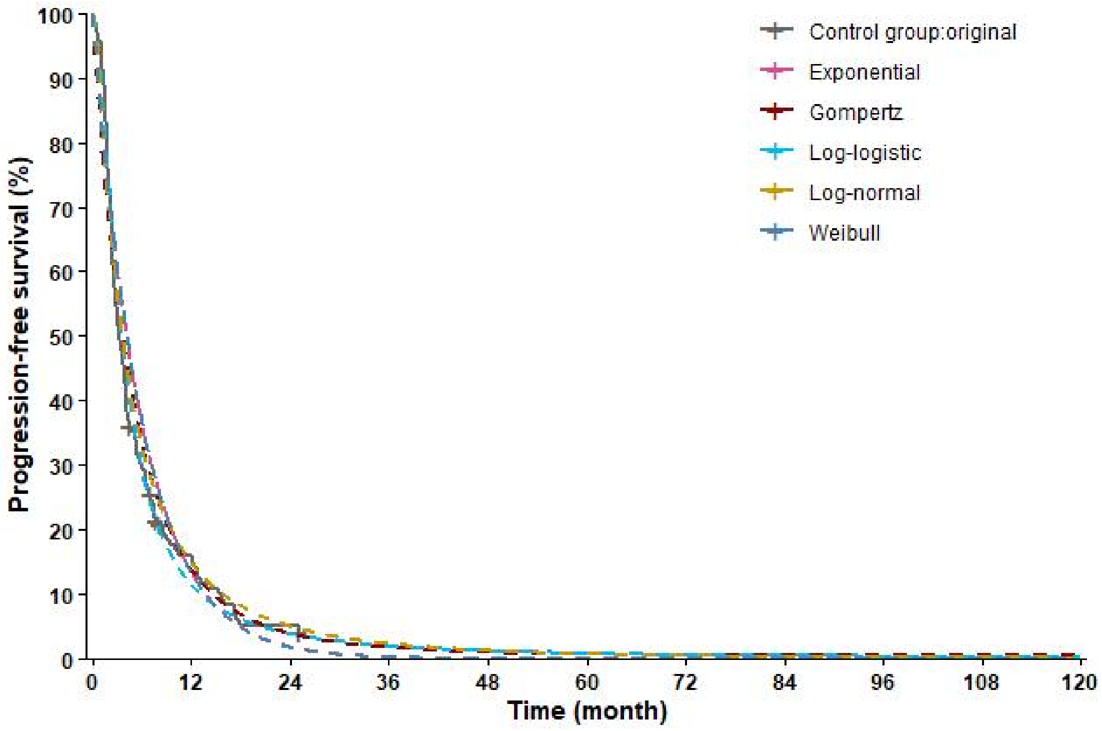

Supplementary Figure S6. Extrapolation of the PFS curves for the overall population to the control group.

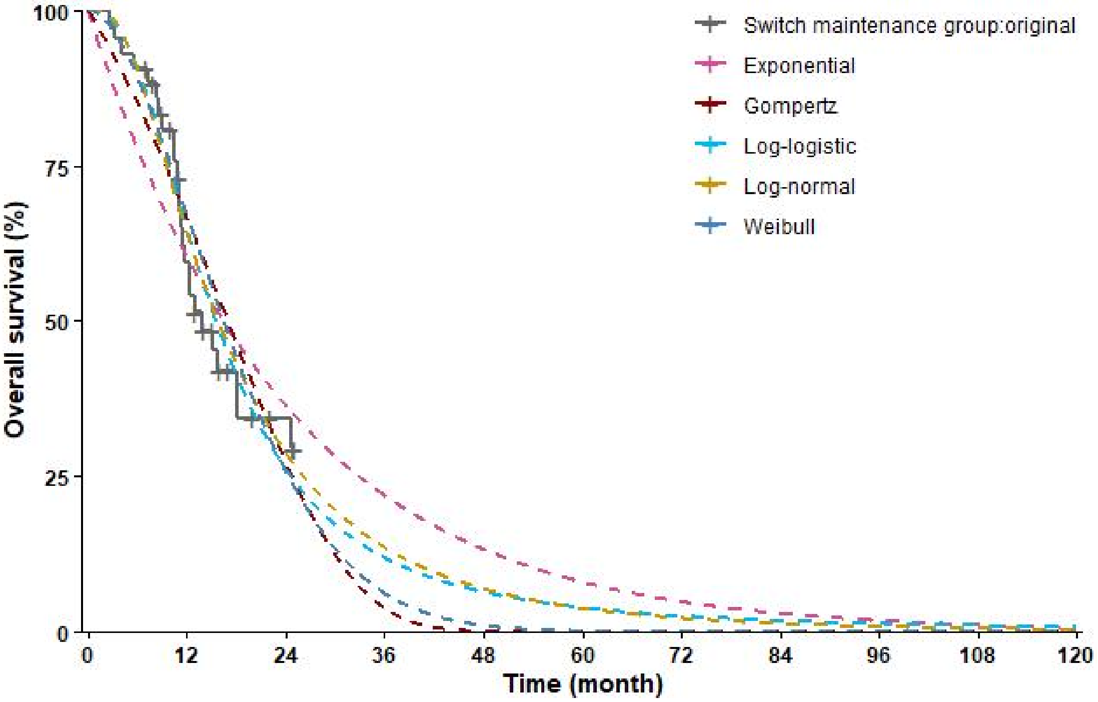

Supplementary Figure S7. Extrapolation of the OS curves for the PDL1-CPS⩾5 population to the switch maintenance group.

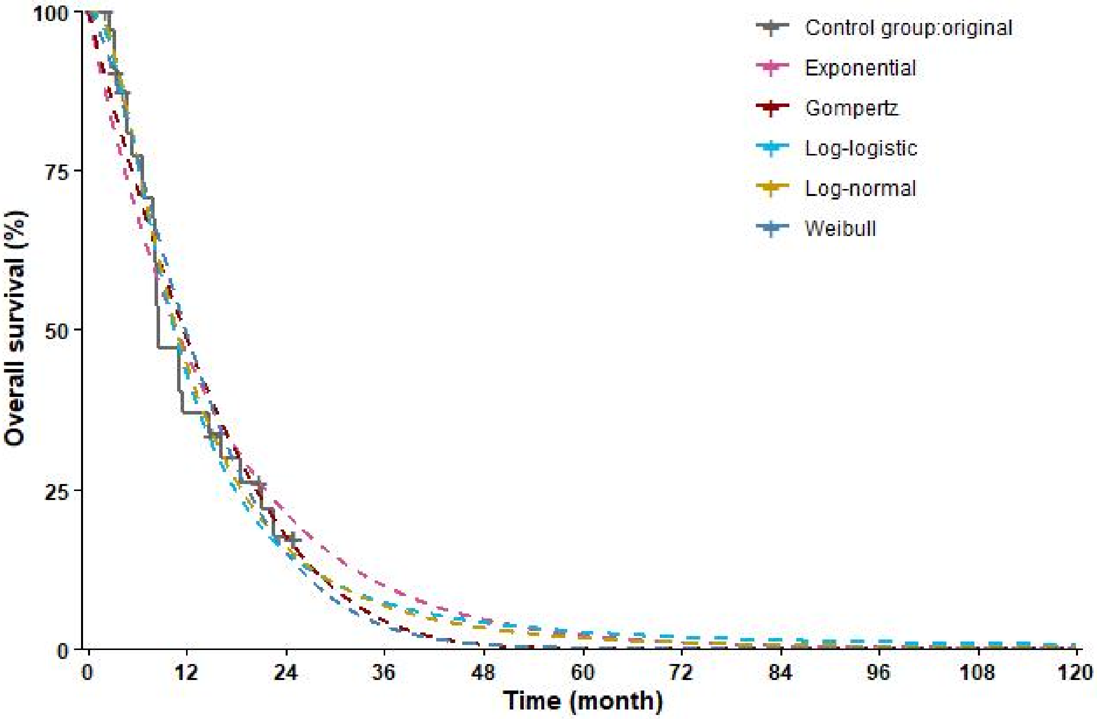

Supplementary Figure S8. Extrapolation of the OS curves for the PDL1-CPS≥5 population to the control group.

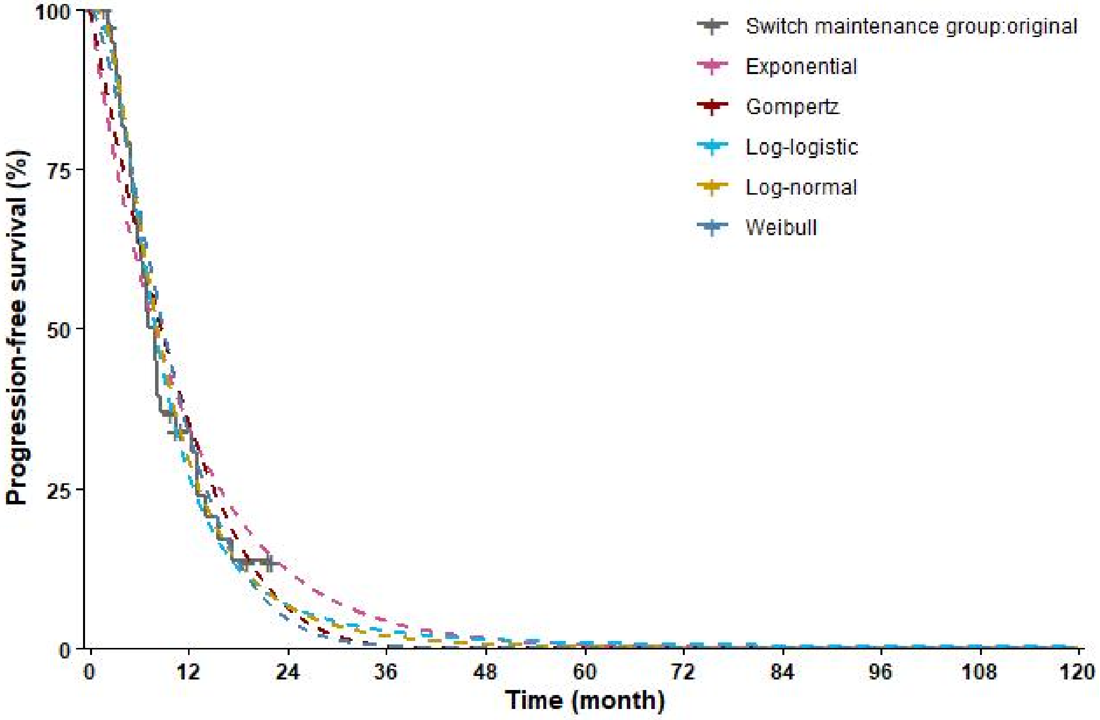

Supplementary Figure S9. Extrapolation of the PFS curves for the PDL1-CPS≥5 population to the switch maintenance group.

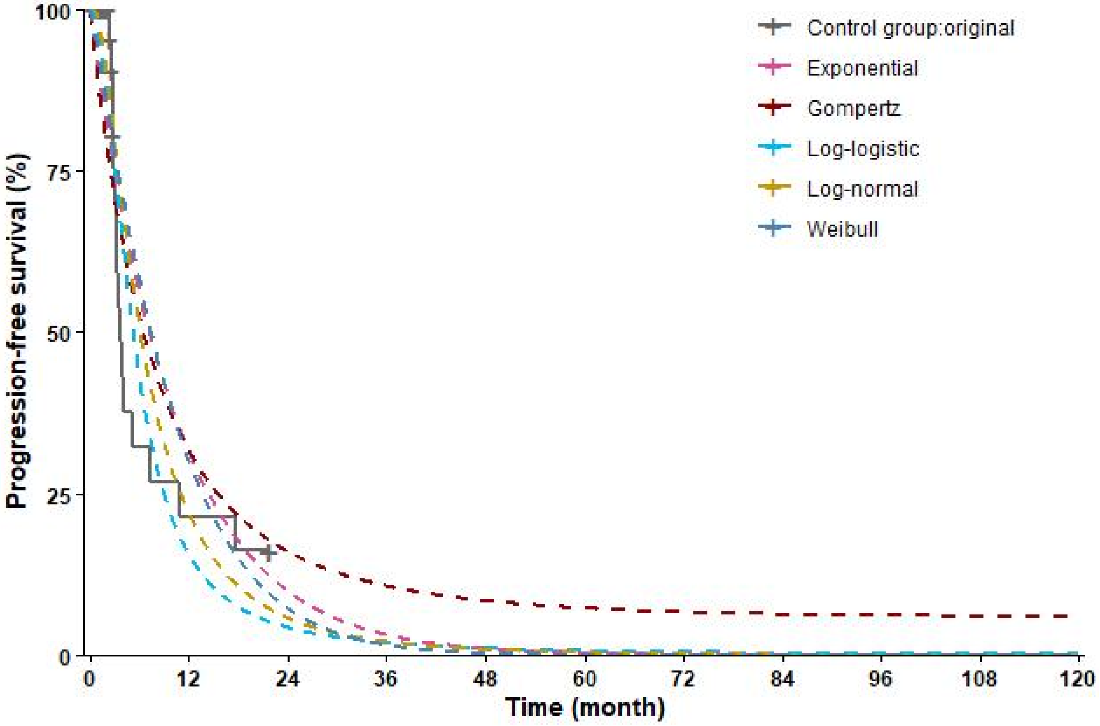

Supplementary Figure S10. Extrapolation of the PFS curves for the PDL1-CPS ≥5 population to the switch maintenance group.
